# Supplementary material for: Antibacterial Performance of a Mussel-Inspired Polydopamine-Treated Ag/Graphene Nanocomposite Material
Source: Materials (Basel). 2019 Oct 15;12(20):3360. doi: 10.3390/ma12203360 (PMC6829421; doi:10.3390/ma12203360)
Supplement: Supplementary file 1 [file materials-12-03360-s001.pdf]

materials

MDPI

Supplymentary Materials

Antibacterial Performance of a Mussel-Inspired Polydopamine-Treated Ag/Graphene Nanocomposite Material

Jianming Liao, Shuaiming He, Shasha Guo, Pengcheng Luan, Lihuan Mo * and Jun Li *

State Key Laboratory of Pulp and Paper Engineering, South China University of Technology, Guangzhou 510641, China; feliaojm_1992@mail.scut.edu.cn (J.L); he.shuaiming@gmail.com (S.H.); feshasha.guo@mail.scut.edu.cn (S.G.); luanpc1991@gmail.com (P.L.)

* Correspondence: lhmo@scut.edu.cn (L.M); ppjunli@scut.edu.cn (J.L.); Tel.: +86-153-2334-0152 (L.M); Tel.: +86-135-0301-2206 (J.L.)

Received: 22 August 2019; Accepted: 12 October 2019; Published: 15 October 2019

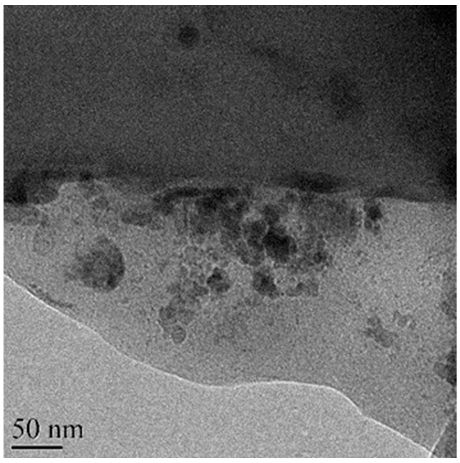

Figure S1. The TEM image of Ag-PDA-RGO.

© 2019 by the authors. Submitted for possible open access publication under the terms and conditions of the Creative Commons Attribution (CC BY) license (http://creativecommons.org/licenses/by/4.0/).
